# Regulation of Tissue LC-PUFA Contents, Δ6 Fatty Acyl Desaturase (FADS2) Gene Expression and the Methylation of the Putative FADS2 Gene Promoter by Different Dietary Fatty Acid Profiles in Japanese Seabass (*Lateolabrax japonicus*)

**DOI:** 10.1371/journal.pone.0087726

**Published:** 2014-01-31

**Authors:** Houguo Xu, Xiaojing Dong, Qinghui Ai, Kangsen Mai, Wei Xu, Yanjiao Zhang, Rantao Zuo

**Affiliations:** 1 Key Laboratory of Aquaculture Nutrition and Feed (Ministry of Agriculture) & Key Laboratory of Mariculture (Ministry of Education), Ocean University of China, Qingdao, China; 2 Key Laboratory of Chinese Ministry of Agriculture for Sustainable Utilization of Marine Fisheries Resources, Yellow Sea Fisheries Research Institute, Qingdao, China; 3 Key Laboratory of Mariculture and Stock Enhancement in North China's Sea (Ministry of Agriculture), Dalian Ocean University, Dalian, China; Virginia Tech, United States of America

## Abstract

The present study was conducted to evaluate the influences of different dietary fatty acid profiles on the tissue content and biosynthesis of LC-PUFA in a euryhaline species Japanese seabass reared in seawater. Six diets were prepared, each with a characteristic fatty acid: Diet PA: Palmitic acid (C16:0); Diet SA: Stearic acid (C18:0); Diet OA: Oleic acid (C18:1n-9); Diet LNA: α-linolenic acid (C18:3n-3); Diet N-3 LC-PUFA: n-3 LC-PUFA (DHA+EPA); Diet FO: the fish oil control. A 10-week feeding trial was conducted using juvenile fish (29.53±0.86 g). The results showed that Japanese seabass had limited capacity to synthesize LC-PUFA and fish fed PA, SA, OA and LNA showed significantly lower tissue n-3 LC-PUFA contents compared to fish fed N-3 LC-PUFA and FO. The putative gene promoter and full-length cDNA of FADS2 was cloned and characterized. The protein sequence was confirmed to be homologous to FADS2s of marine teleosts and possessed all the characteristic features of microsomal fatty acid desaturases. The FADS2 transcript levels in liver of fish fed N-3 LC-PUFA and FO were significantly lower than those in fish fed other diets except LNA while Diet PA significantly up-regulated the FADS2 gene expression compared to Diet LNA, N-3 LC-PUFA and FO. Inversely, fish fed N-3 LC-PUFA and FO showed significantly higher promoter methylation rates of FADS2 gene compared to fish fed the LC-PUFA deficient diets. These results suggested that Japanese seabass had low LC-PUFA synthesis capacity and LC-PUFA deficient diets caused significantly reduced tissue n-3 LC-PUFA contents. The liver gene expression of FADS2 was up-regulated in groups enriched in C16:0, C18:0 and C18:1n-9 respectively but not in the group enriched in C18:3n-3 compared to groups with high n-3 LC-PUFA contents. The FADS2 gene expression regulated by dietary fatty acids was significantly negatively correlated with the methylation rate of putative FADS2 gene promoter.

## Introduction

Fish have been considered as the most important source of long-chain polyunsaturated fatty acids (LC-PUFA) for both human consumption and as feed ingredients for farmed fish and other food animals [1], [2], [3]. Although aquaculture is expected to meet much of the growing consumer demand for LC-PUFA, it substantially depends on fish oils deriving from wild-catch fish as source of LC-PUFA [4], [5]. Thus, to reduce the dependence of LC-PUFA production on wild fisheries, as well as to find ways to use marine LC-PUFA more economically and efficiently, alternative oils such as animal oils and vegetable oils should be used in aquaculture and the effects of alternative oils on LC-PUFA depositions and possible LC-PUFA synthesis in cultured fish need to be evaluated.

It is well documented that freshwater teleosts have a capacity to desaturate-elongate 18C fatty acids into 20-22C LC-PUFAs and this capacity is assumed to be low or absent in marine fish [6], [7]. Anadromous fish such as salmon appear to have desaturase profile of freshwater fish [8], [9] but euryhaline fish commonly show varied capacity to synthesize LC-PUFAs according to the ambient salinity [10], [11], [12], [13]. Japanese seabass *Lateolabrax japonicas*, which is one of the most commercially valuable cultured species in Asia and recorded the largest annal production (more than 132 000 ton) among marine and euryhaline cultured species in China, also has the ability to adapt to a wide range of salinity. Up to date, no information has been reported about either the LC-PUFA synthesis capacity or its nutritional regulation in Japanese seabass. The present study was conducted to investigate the fatty acid desaturation in Japanese seabass juveniles reared in seawater as well as to evaluate its regulation by different dietary fatty acid profiles.

In the biosynthesis of LC-PUFA, Δ6 desaturase (FADS2), which catalyzes the first desaturation step, plays a vital role and thus was commonly used to indicate the LC-PUFA synthesis capacity in fish [14], [15]. The activity of FADS2 in marine and euryhaline fish and its nutritional regulation have been investigated in several species [10], [14], [16], [17], [18], [19], [20], [21], [22], [23], [24]. In recent years, molecular biological approaches are being used more and more frequently to study the mechanisms involved in the nutritional regulation of FADS2 [25]. The gene expression of FADS2 and its nutritional regulation have been reported in several studies on euryhaline or marine fish. However, further mechanisms in the regulation of FADS2 gene expression, especially by dietary fatty acids, were rarely studied in fish up to date. In mammal, transcription factors such as peroxisome proliferator activated receptors (PPARs) and sterol regulatory element binding protein-1 (SREBP-1), which are the main transcription factors regulating the gene expression of fatty acid elongase and desaturase [26], [27], have been reported to be regulated by dietary fatty acids [28], [29], [30], [31], [32]. Previous studies on rodents suggested that the transcription of FADS2 could also be regulated by diets through the alteration in the methylation status of gene promoter [33], [34], which usually occurs in CpG dinucleotides and is one of the major epigenetic mechanisms implicated in the regulation of gene transcription, normally associated with gene inactivation and silencing of gene expression. Up to date, little information was available about the effects of dietary fatty acid profiles on the promoter methylation status of desaturase genes [35], [36]. Thus, in the present study, the effects of different fatty acid profiles on the methylation status of FADS2 putative promoter were investigated in Japanese seabass, along with the investigation on effects of dietary fatty acids on FADS2 transcription.

In the present study, a 10-week feeding experiment was conducted on juvenile Japanese seabass, *Lateolabrax japonicus* to comprehensively compare the effects of different dietary fatty acid profiles i.e., the LC-PUFA deficient fatty acid profiles and the n-3 LC-PUFA rich profiles, on tissue n-3 LC-PUFA contents, FADS2 mRNA expression, and the methylation status of putative FADS2 promoter, as well as to evaluate the correlations among these effects. Also, in this study the cDNA and putative promoter of Δ6 fatty acyl desaturase (FADS2) from Japanese seabass was first cloned and characterized.

## Materials and Methods

### Ethics statement

The present study was carried out in strict accordance with the recommendations in the Guide for the Use of Experimental Animals of Ocean University of China. The protocols for animal care and handling used in this study were approved by the Institutional Animal Care and Use Committee of Ocean University of China (Permit Number: 20001001). Before sacrificing and handling, experimental fish were first anesthetized with eugenol (1∶10,000) (Shanghai Reagent, China), and all efforts were made to minimize suffering.

### Experimental diets

Six experimental diets with similar proximate compositions (42% crude protein and 11% lipid) were formulated, differing only in the lipid source (Table 1). Five different lipid sources, tripalmitin (Shanghai Zhixin chemical Co., Ltd., Shanghai, China), tristearin (HUDONG article of everyday use Co., Ltd., Jiaxing, China), rapeseed oil, blended oil of linseed oil and perilla oil (linseed oil: perilla oil  = 3∶1), and n-3 LC-PUFA enriched oil (with 37.1% DHA and 20.8% EPA of total fatty acids (TFA); in the form of triglyceride; HEBEI HAIYUAN Health biological Science and Technology Co., Ltd., Cangzhou, China), were supplemented separately to the basal diet at a ratio of 10% to obtain five diets with different characteristic fatty acids, respectively: Diet PA, palmitic acid (C16:0); Diet SA, stearic acid (C18:0); Diet OA, oleic acid (C18:1n-9); Diet LNA, α-linolenic acid (C18:3n-3); and Diet N-3 LC-PUFA, n-3 LC-PUFA (C22:6n-3+C20:5n-3). The sixth diet was formulated with 10% fish oil (Diet FO) supplemented to the basal diet. The fatty acid profiles analyzed by high-performance gas chromatography (HP6890, USA) were presented in Table 2.

**Table 1:** Formulation (%) and proximate composition (%) of the experiment diets.

Gradients	PA	SA	OA	LNA	N-3 LC-PUFA	FO
Defatted fish meal^1^	26.00	26.00	26.00	26.00	26.00	26.00
Soybean meal^1^	34.00	34.00	34.00	34.00	34.00	34.00
Wheat meal	25.91	25.91	25.91	25.91	25.91	25.91
Tripalmitin^2^	10.00					
Tristearin^3^		10.00				
Rapeseed oil			10.00			
C18:3n-3 enriched oil 4				10.00		
n-3 LC-PUFA enriched oil^5^					10.00	
Fish oil						10.00
Mineral premix^6^	2.00	2.00	2.00	2.00	2.00	2.00
Vitamin premix^7^	1.60	1.60	1.60	1.60	1.60	1.60
Mold inhibitor^8^	0.10	0.10	0.10	0.10	0.10	0.10
Attractant^9^	0.30	0.30	0.30	0.30	0.30	0.30
Ethoxyquin	0.05	0.05	0.05	0.05	0.05	0.05
Yttria	0.04	0.04	0.04	0.04	0.04	0.04
*Proximate analysis*						
Crude protein	41.67	41.54	42.17	42.29	42.25	42.18
Crude lipid	11.36	11.16	10.93	10.89	11.26	11.51
Ash	11.06	10.47	11.16	10.46	10.84	11.25

1 Defatted fish meal: 75.6% crude protein, 1.8% crude lipid; soybean meal: 51.7% crude protein, 2.0% crude lipid; of dry matter.

2 Shanghai Zhixin chemical Co., Ltd., Shanghai, China.

3 HUDONG article of everyday use Co., Ltd., Jiaxing, China.

4 Blended oil of linseed oil and perilla oil (Linseed oil: Perilla oil  = 3:1).

5 n-3 LC-PUFA enriched oil: DHA content, 37.1% of total fatty acid (TFA); EPA content, 20.8% of TFA; both in the form of triglyceride; HEBEI HAIYUAN Health biological Science and Technology Co., Ltd., Cangzhou, China.

6 Mineral premix (mg or g/kg diet): MgSO_4_·7H_2_O, 1200 mg; CuSO_4_·5H_2_O, 10 mg; ZnSO_4_·H_2_O, 50 mg; FeSO_4_·H_2_O, 80 mg; MnSO_4_·H_2_O, 45 mg; CoCl_2_·6H_2_O (1%), 50 mg; NaSeSO_3_·5H_2_O (1%), 20 mg; Ca(IO_3_)_2_·6H_2_O (1%), 60 mg; CaH_2_PO_4_·H_2_O, 10 g; zoelite, 8.485 g.

7 Vitamin premix (mg or g/kg diet): thiamin 25 mg; riboflavin, 45 mg; pyridoxine HCl, 20 mg; vitamin B_12_ (1%), 10 mg; vitamin K_3_, 10 mg; inositol, 800 mg; pantothenic acid, 60 mg; niacin, 200 mg; folic acid, 20 mg; biotin (2%), 60 mg; retinol acetate, 32 mg; cholecalciferol, 5 mg; alpha-tocopherol (50%), 240 mg; ascorbic acid, 2000 mg; choline chloride (50%), 4000 mg; wheat middling, 8.47 g.

8 Mold inhibitor: contained 50% calcium propionic acid and 50% fumaric acid.

9 Attractant: glycine and betaine.

**Table 2:** Fatty acid profiles of experimental diets (of total fatty acid)^1^.

Fatty acid	PA	SA	OA	LNA	N-3 LC-PUFA	FO
C 14: 0	0.79	1.94	0.29	0.38	0.65	4.13
C 16: 0	56.30	25.64	7.69	10.08	6.17	20.92
C 18: 0	18.60	55.78	3.62	4.99	7.13	14.25
∑SFA^2^	75.69	83.36	11.60	15.45	13.95	39.31
C 16: 1n-7	1.09	0.32	tr	tr	2.59	4.89
C 18: 1n-9	10.03	2.62	41.06	29.00	7.05	8.96
C 20: 1n-9	1.36	0.89	6.64	1.11	2.36	4.40
C 22: 1n-11	0.64	0.59	tr	0.22	0.57	4.93
C 22: 1n-9	nd	nd	15.97	nd	nd	nd
∑MUFA^3^	13.12	4.42	63.66	30.33	12.57	23.18
C 18: 2n-6	6.37	5.88	16.74	14.81	14.91	10.69
C 20: 4n-6	0.07	0.08	0.10	0.10	2.73	0.73
∑n-6^4^	6.54	6.32	16.85	14.91	17.86	11.64
C 18: 3n-3	0.80	1.04	5.48	35.38	2.17	2.13
C 18: 4n-3	0.11	0.09	0.11	0.15	0.93	1.36
C 20: 5n-3	0.74	0.63	0.67	0.87	15.19	6.03
C 22: 5n-3	0.08	0.07	0.08	0.09	1.19	0.70
C 22: 6n-3	0.87	0.84	0.83	1.18	28.80	9.12
∑n-3^5^	2.60	2.67	7.18	37.68	48.28	19.33
∑PUFA^6^	9.15	9.00	24.02	52.59	66.14	30.97
∑n-3LC-PUFA	1.69	1.55	1.58	2.15	45.18	15.84
∑n-3/∑n-6	0.40	0.42	0.43	2.53	2.70	1.66
C18: 1n-9/∑n-3	3.85	0.98	5.72	0.77	0.15	0.46

1 Some fatty acids, of which the contents are minor, trace amount or not detected, such as C22: 0, C24: 0, C14: 1, C20: 2n-6, C20:3n-6, were not listed in the table. tr: trace; nd: non-detected.

2 SFA: saturated fatty acid.

3 MUFA: monounsaturated fatty acid.

4 n-6: n-6 unsaturated fatty acid.

5 n-3: n-3 unsaturated fatty acid.

6 PUFA: polyunsaturated fatty acid.

Ingredients were ground into fine powder through 200 µm mesh. All ingredients were thoroughly mixed with the oils, and water was added to produce stiff dough. The dough was then pelleted with an experimental feed mill and dried for 12 h in a ventilated oven at 45°C. After drying, the diets were broken up and sieved into proper pellet size (5.0×5.0 mm), and were stored at −15°C until used.

### Feeding procedures

Japanese seabass (*Lateolabrax japonicus*) were obtained from a commercial farm in Ningbo, China. Prior to the start of the experiment, the juveniles were reared in floating sea cages (3.0×3.0×3.0 m), and fed a low-lipid commercial diet for 2 weeks to acclimate to the experimental conditions.

At the onset of the feeding trial, the fish were fasted for 24 h and weighed after being anesthetized with eugenol (1∶10, 000) (Shanghai Reagent, China). Fish of similar sizes (29.53±0.86 g) were randomly distributed into 18 sea cages (1.5×1.5×2.0 m) and each cage was stocked with 30 fish. Each diet was randomly assigned to triplicate cages. Fish were hand-fed to apparent satiation twice daily (05:00 and 17:00). The feeding trial lasted for 10 weeks. During the experimental period, the temperature ranged from 23.5 to 28.5°C, salinity from 29‰ to 32‰ and the dissolved oxygen was approximately 6∼7 mg l^−1^. At the end of the experiment, the fish were fasted for 24 h before harvest. Samples of liver, muscle, gut, and serum from 5 fish each cage were obtained, frozen with liquid nitrogen and stored at −80°C until use.

### Assay of fatty acid composition

The fatty acid contents of diets and fish tissues were determined as described by Mourente et al. [37] with minor modifications using gas chromatography (HP6890, USA). Results were expressed as percentage of total fatty acids.

### Cloning of full-length putative FADS2 cDNA

Liver of Japanese seabass was grinded in liquid nitrogen. Total RNA was extracted using TransZol (TransGen, China) and then electrophoresed on 1.5% agarose gel to test the quality and integrity followed by concentration determination with NanoDrop®ND-1000 (Nano-Drop Technologies, Wilmington, DE, USA). Then, 1 µg total RNA was subjected to TransScript ™ One-Step gDNA Removal and cDNA Synthesis SuperMix (TransGen China) in 20 µl volume for reverse transcription and DNA erasure. PCR primers to amplify internal fragments of FADS2 cDNA were designed based on the highly conserved regions from genes of other fish available in GeneBank database and synthesized by Biosune (Shanghai, China). PCR amplifications using primer FADS2-F and FADS2-R (Table 3) and Taq DNA Polymerase (Takara) were performed with an initial denaturation at 94°C for 3 min, 35 cycles of denaturation at 57.5°C for 30 s, and extension at 72°C for 1 min, followed by a final extension at 72°C for 10 min.

**Table 3:** Sequences of the primers used in this work.

Primer	Sequence (5′-3′)	Sequence information
FADS2-F (forward)	GGCCACCTGTCTGTCTTCAA	RT primer
FADS2-R (reverse)	GCCACCAGGTGGTAGTTGTG	RT primer
FADS2 -3F (forward)	CAGCGGACACCTCAACTTTC	3′RACE primer
FADS2 -5R1 (reverse)	CATCTGAGTCACCCACACAAACCA	5′RACE primer
FADS2-5R2 (reverse)	CCGCTGAACCAGTCGTTGAAGAGGGA	5′RACE primer
FADS2-RTF (forward)	CCGTCGCGACTGGGTGGATATG	Real-time primer
FADS2-RTR (reverse)	CAGTCCCGGTGCTTCTCGTGG	Real-time primer
β-actin-F (forward)	CAACTGGGATGACATGGAGAAG	β-actin Real-time primer
β-actin-R (reverse)	TTGGCTTTGGGGTTCAGG	β-actin Real-time primer
SP1 (reverse)	CGTGGCATCCTCTCCAGAATAGTG	Promoter primer
SP2 (reverse)	ACTGGTCGTTCCTGCTGCGGT	Promoter primer
SP3 (reverse)	AGCTGACCCCCGCCTCCCAT	Promoter primer
BF1 (forward)	TTTAGTTTAAAGTTGATTTAAATAGAT	BSP primer
BR1 (reverse)	ACAAAAAAACTACTAAACCCCAAAC	BSP primer
BF2 (forward)	AGTGGTTTAGGATATATTTATTGTG	BSP primer
BR2 (reverse)	TTAAAAAAATTACACCATCTTACCC	BSP primer

The 3′and 5′ ends of FADS2 cDNA were determined by the RACE-PCR method as detailed in the manufacturer's protocol. Three gene-specific primers were designed based on the known sequences of the internal fragment of FADS2 cDNA singlet to clone the 3′- and 5′-end of cDNA by rapid amplification of cDNA ends (RACE) using the SMARTer™ RACE cDNA Amplification Kit (Clontech, USA) (Table 3). The 3′- and 5′-end cDNA templates were synthesized according to the user's manual. For 3′ RACE, only one PCR round was performed using primer FADS2-3F (Table 3), and for 5′RACE, the first and the nested PCR rounds were performed using primers FADS2-5R1 and FADS2-5R2 (Table 3).

The PCR products were run on a 1.5% agarose gel, and then purified using Aqua-SPIN Gel Extraction Mini Kit (Biowatson, Shanghai). The purified PCR products were cloned into pEASY-T1 vector (TransGen), and the recombinant vector was transformed into competent Trans1-T1 cells (TransGen). The inserted PCR fragments were sequenced on an ABI3730XL.

### Real-time quantitative reverse transcriptase-polymerase chain reaction (qRT-PCR) analysis

The mRNA expression pattern of the FADS2 transcript in various tissues (kidney, skin, muscle, gill, spleen, eye, stomach, intestine, blood, brain and heart) and liver of Japanese seabass fed different diets were measured by real-time fluorescent quantitative PCR (qRT-PCR) using β-actin (GeneBank accession number: HE577671.1) as reference gene. Specific primers for FADS2 (FADS2-RTF and FADS2-RTR) and β-actin (β-actin-F and β-actin-R) were designed using Primer 5.0 software based on the partial cDNA sequences obtained as described previously (Table 3). The real-time PCR was carried out in a quantitative thermal cycler (Mastercycler eprealplex, Eppendorf, German) in a final volume of 25 µl containing 2×SYBR Green Real-time PCR Master Mix (TaKaRa, Japan), primer pairs and cDNA. The program was 95°C for 30 s followed by 35 cycles of 95°C for 5 s, 58°C for 15 s and 72°C for 20 s. Melting curve (1.85°C increment/min from 58°C to 95°C) was performed after the amplification phase for confirmation. Each sample was run in triplicate. The gene expression levels of putative FADS2 in liver of Japanese seabass fed different diets were studied by qRT-PCR method: 2^−ΔΔCT^ [38].

### Cloning of FADS2 promoters and determination of the methylation status

#### DNA purification and bisulphate modification

DNA was prepared from liver that had been snap-frozen on harvesting and kept in liquid nitrogen. A ratio of >1.5 from OD measurements at 260/280 nm was required for efficient conversion of unmethylated cytosine residues into uracil sodium bisulphate treatment. DNA with this level of purity was obtained using the SQ Tissue DNA Kit (OMEGA), according to the manufacturer's instructions.

Sodium bisulphate modification was carried out with the EZ DNA Methylation-Gold kit (ZYMO Research) in accordance with the manufacturer's instructions, in order to convert the unmethylated cytosines to uridines and leave the methylated cytosines unchanged.

#### Cloning of FADS2 promoters

Based on the cloned FADS2 mRNA of *Lateolabrax japonicas* (JX678842.1), three reverse primers, SP1, SP2, and SP3 (Table 3) were used in three rounds of polymerase chain reaction (PCR) for *Lateolabrax japonicas* FADS2 promoter cloning, respectively. The nested PCR (three rounds) was performed to isolate FADS2 promoter from genomic DNA using the Genome Walking Kit (TaKaRa Biotechnology) according to the manufacturer's instructions. The reverse primer was AP4 in all PCR reactions, which was supplied in the Kit. For FADS2 promoter cloning, the conditions for the first round of PCR were 1 cycle (94°C for 1 min, 98°C for 1 min), 5 cycles (94°C for 30 s, 62°C for 1 min, and 72°C for 2 min), 1 cycle (94°C for 30 s, 25°C for 3 min, and 72°C for 2 min), 15 cycles (94°C for 30 s, 62°C for 1 min, and 72°C for 2 min; 94°C for 30 s, 62°C for 1 min, and 72°C for 2 min; 94°C for 30 s, 44°C for 1 min, and 72°C for 2 min), and 1 cycle (72°C for 10 min). The conditions for the second round of PCR were 15 cycles (94°C for 30 s, 61°C for 1 min, and 72°C for 2 min; 94°C for 30 s, 61°C for 1 min, and 72°C for 2 min; 94°C for 30 s, 44°C for 1 min, and 72°C for 2 min) and 1 cycle (72°C for 10 min). The conditions for the third round of PCR were 15 cycles (94°C for 30 s, 63°C for 1 min, and 72°C for 2 min; 94°C for 30 s, 63°C for 1 min, and 72°C for 2 min; 94°C for 30 s, 44°C for 1 min, and 72°C for 2 min) and 1 cycle (72°C for 10 min).

#### Bisulphite genomic sequencing PCR (BSP)

The amplification of bisulfite-modified DNA for FADS2 gene promoter was performed by PCR with primers that were specific for either methylated or unmethylated FADS2 sequences. Primers used to amplify the methylated FADS2 gene were BF1, BR1, BF2 and BR2 (Table 3).

One and a half microliter bisulfate modified DNA from each sample were subjected to PCR analysis in a 40 µl volume containing 20 µl 2×PCR Master Mix, 1 µl MgSO_4_, and 0.5 µl primer. The reaction mixture was preheated at 95°C for 5 min and the amplified conditions were 16 cycles (95°C for 30 s, 62°C for 35 s, and 72°C for 1 min) and 30 cycles (95°C for 30 s, 57°C for 30 s, and 72°C for 1 min). The PCR products were then cloned into the pEASY-T1 vector (TransGen), followed by sequencing analysis (after the cloning, 10 clones from each sample were randomly selected for DNA sequencing).

### Statistical analysis

All data were subjected to one-way analysis of variance and correlation analysis where appropriate in SPSS 16.0 (SPSS Inc., USA) for Windows. Differences between the means were tested by Tukey's multiple range test. The level of significance was chosen at *P*<0.05 and the results were presented as means ± S.E.M. (standard error of the mean).

Similarity searches of the sequenced cDNA and promoter of FADS2 were done by blastn (www.ncbi.nlm.nih.gov/blast/). The multiple-sequence alignments were performed using ClustalW. Phylogenetic analyses were carried out based on amino acid sequences using the neighbour-joining method, and the trees were constructed using MEGA 4.1. MethPrimer (http://www.urogene.org/methprimer) was used to identify the CpG islands. For the data obtained from the BSP PCR-based sequencing analysis, the percentage of methylation in a given sample was calculated as the height of the “C” peak divided by the sum of the height of “C” + “T”.

## Results

### Fatty acid composition

#### Diets

The fatty acid analysis of the diets showed that the content of the characteristic fatty acid of each diet was as follows (% TFA): Diet PA: 56.30% C16:0; Diet SA: 55.78% C18:0; Diet OA: 41.06% C18:1n-9; Diet LNA: 35.38% C18:3n-3; Diet N-3 LC-PUFA: 28.80% DHA and 15.19% EPA (Table 2). The control diet (FO) had a typical fatty acid profile of fish oil (20.92% C16:0, 14.25% C18:0, 8.96% C18:1n-9, 10.69% C18:2n-6, 0.73% C20:4n-6, 2.13% C18:3n-3, 6.03% EPA, and 9.12% DHA). Diet PA, SA, OA, and LNA had obviously lower n-3 LC-PUFA contents (1.69%, 1.55%, 1.58%, and 2.15%, respectively) than Diet N-3 LC-PUFA (45.18%) and FO (15.84%).

#### Fish tissues

**Figure 1:**
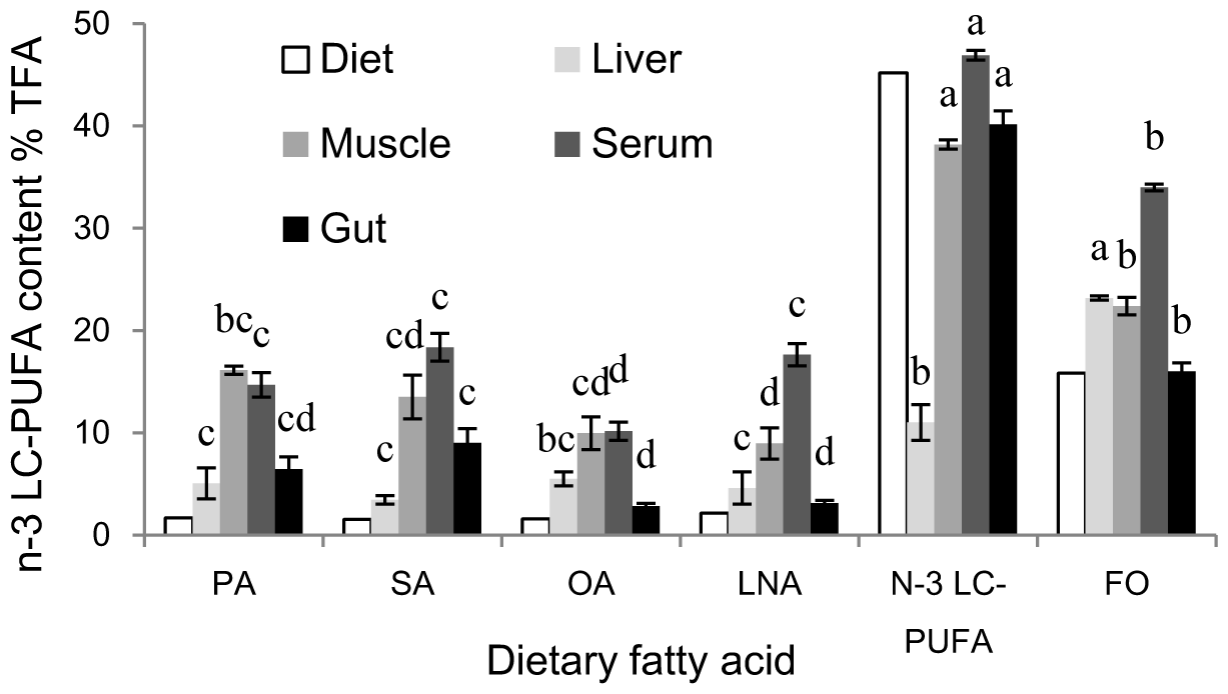
Contents of n-3 LC-PUFA in diets and tissues of experimental fish. Values (means ± S.E.M.) in bars that do not have the same letter are significantly different (*P*<0.05) among treatments. The fatty acid contents were expressed as percentage of total fatty acids.

Fish fed PA, SA, OA and LNA showed significantly (*P*<0.01) lower n-3 LC-PUFA contents in muscle, liver, serum, and gut compared to fish fed N-3 LC-PUFA and FO (Tables 4, 5, 6, and 7; Figure 1). The n-3 LC-PUFA contents in group N-3 LC-PUFA was significantly higher (*P*<0.01) than those in group FO in all tested tissues except liver whereas it followed an opposite pattern in liver. The n-3 LC-PUFA contents in muscle and gut of fish fed OA and LNA was lower compared to fish fed PA and SA while fish fed OA showed the lowest n-3 LC-PUFA content in serum among dietary treatments.

**Table 4:** Liver fatty acid profiles of juvenile Japanese seabass fed diets with different fatty acid profiles (of total fatty acid)^1^ (means ± S.E.M., n = 3)*.

Fatty acid	PA	SA	OA	LNA	N-3 LC-PUFA	FO	*P* value
C 14: 0	1.98±0.14^b^	2.55±0.04^a^	1.09±0.06^c^	1.13±0.16^c^	1.70±0.11^b^	2.10±0.04^ab^	0.000
C 16: 0	20.93±0.76^a^	20.53±1.53^a^	10.32±0.24^c^	12.38±1.37^bc^	22.98±2.14^a^	17.28±0.24^ab^	0.000
C 18: 0	15.21±0.90^b^	18.56±0.55^ab^	21.31±1.60^a^	22.43±0.47^a^	8.76±1.08^c^	4.45±0.28^c^	0.000
∑SFA^2^	38.12±1.23^ab^	41.65±2.06^a^	32.72±1.51^b^	35.94±1.19^ab^	33.43±3.28^ab^	23.82±0.53^c^	0.000
C 16: 1n-7	11.05±0.86^a^	11.27±0.40^a^	3.56±0.22^c^	4.19±0.88^bc^	4.98±0.38^bc^	6.44±0.22^b^	0.000
C 18: 1n-9	28.69±1.26^a^	24.71±0.68^abc^	22.80±1.24^bc^	20.63±1.29^c^	26.40±1.44^ab^	23.50±0.60^abc^	0.002
C 20: 1n-9	1.22±0.16^c^	1.05±0.07^c^	4.84±0.06^a^	1.45±0.08^c^	3.57±0.49^b^	2.65±0.16^b^	0.000
C 22: 1n-11	0.13±0.03^c^	0.12±0.01^c^	tr	0.10±0.02^c^	0.82±0.02^b^	0.95±0.02^a^	0.000
C 22: 1n-9	nd	nd	4.03±0.35	nd	nd	nd	
∑MUFA^3^	41.08±0.76^a^	37.14±1.04^ab^	35.23±1.42^ab^	26.37±2.16^c^	35.78±2.13^ab^	33.55±0.90^b^	0.000
C 18: 2n-6	6.05±0.76^cd^	5.13±0.37^d^	16.84±0.39^a^	12.32±1.04^ab^	9.74±1.92^bc^	8.76±0.09^bcd^	0.000
C 20: 4n-6	0.30±0.09^b^	0.21±0.03^b^	0.28±0.04^b^	0.21±0.08^b^	1.44±0.19^a^	1.43±0.02^a^	0.000
∑n-6^4^	6.35±0.72^cd^	5.34±0.36^d^	17.12±0.35^a^	12.54±1.11^ab^	11.19±2.10^bc^	10.19±0.08^bcd^	0.000
C 18: 3n-3	0.83±0.36^c^	1.31±0.35^c^	2.81±0.11^b^	15.45±0.33^a^	0.80±0.10^c^	1.39±0.09^c^	0.000
C 18: 4n-3	0.30±0.04^c^	0.62±0.03^bc^	0.94±0.05^b^	3.27±0.21^a^	0.37±0.11^c^	0.57±0.01^bc^	0.000
C 20: 5n-3	0.78±0.23^c^	0.56±0.08^c^	0.78±0.11^c^	0.69±0.21^c^	2.32±0.47^b^	4.55±0.14^a^	0.000
C 22: 5n-3	0.47±0.14^b^	0.34±0.04^b^	0.54±0.04^b^	0.47±0.15^b^	0.87±0.26^b^	1.91±0.12^a^	0.000
C 22: 6n-3	3.81±1.15^bc^	2.55±0.29^c^	4.17±0.53^bc^	3.44±1.22^c^	7.82±1.03^b^	16.72±0.25^a^	0.000
∑n-3^5^	6.19±1.66^bc^	5.38±0.20^c^	9.25±0.55^bc^	23.33±1.87^a^	12.19±1.74^b^	25.14±0.27^a^	0.000
∑PUFA^6^	12.54±1.37^c^	10.72±0.48^c^	26.37±0.21^ab^	35.87±2.96^a^	23.37±3.84^b^	35.33±0.30^a^	0.000
∑n-3 LC-PUFA	5.06±1.52^c^	3.45±0.41^c^	5.50±0.68^bc^	4.60±1.58^c^	11.01±1.75^b^	23.18±0.20^a^	0.000
∑n-3/∑n-6	1.04±0.35^b^	1.01±0.06^b^	0.54±0.04^b^	1.87±0.05^a^	1.11±0.05^b^	2.47±0.03^a^	0.000
C18: 1n-9/∑n-3	5.61±1.82^a^	4.61±0.19^ab^	2.48±0.22^abc^	0.90±0.12^c^	2.28±0.41^abc^	0.94±0.03^bc^	0.003

1 Some fatty acids, of which the contents are minor, trace amount or not detected, such as C22: 0, C24: 0, C14: 1, C20: 2n-6, C20:3n-6, were not listed in the table. *Values in the same row with no common superscripts letters are significantly different (*P*<0.05). tr: trace; nd: non-detected.

2 SFA: saturated fatty acid.

3 MUFA: monounsaturated fatty acid.

4 n-6: n-6 unsaturated fatty acid.

5 n-3: n-3 unsaturated fatty acid.

6 PUFA: polyunsaturated fatty acid.

**Table 5:** Muscle fatty acid profiles of juvenile Japanese seabass fed diets with different fatty acid profiles (of total fatty acid)^1^ (means ± S.E.M., n = 3)*.

Fatty acid	PA	SA	OA	LNA	N-3 LC-PUFA	FO	*P* value
C 14: 0	1.25±0.09^bc^	1.71±0.12^b^	0.79±0.07^cd^	0.63±0.03^d^	0.89±0.08^cd^	2.93±0.17^a^	0.000
C 16: 0	22.30±0.55^a^	19.11±0.12^b^	12.79±0.46^c^	12.84±0.30^c^	13.00±0.45^c^	18.54±0.17^b^	0.000
C 18: 0	5.79±0.38^ab^	5.64±0.46^ab^	3.68±0.30^c^	5.95±0.30^a^	4.22±0.31^bc^	4.29±0.29^bc^	0.001
∑SFA^2^	29.34±0.32^a^	26.46±0.24^b^	17.26±0.71^d^	19.42±0.58^c^	18.11±0.29^cd^	25.76±0.30^b^	0.000
C 16: 1n-7	5.86±0.87^a^	7.20±0.97^a^	2.47±0.21^b^	1.84±0.07^b^	2.79±0.41^b^	6.37±0.23^a^	0.000
C 18: 1n-9	27.93±1.03^b^	28.00±1.32^b^	35.97±1.28^a^	27.29±0.83^b^	15.83±0.42^d^	21.32±0.34^c^	0.000
C 20: 1n-9	1.43±0.03^d^	1.63±0.05^cd^	4.53±0.10^a^	1.08±0.01^e^	1.81±0.03^c^	2.99±0.10^b^	0.000
C 22: 1n-9	nd	nd	3.73±0.47	nd	nd	nd	
∑MUFA^3^	35.22±1.84^b^	36.84±2.24^b^	46.70±1.75^a^	30.20±0.90^b^	20.43±0.81^c^	30.68±0.63^b^	0.000
C 18: 2n-6	11.22±0.95^bc^	12.44±0.46^bc^	17.29±1.13^a^	14.25±0.21^ab^	11.10±0.33^bc^	9.76±0.33^c^	0.000
C 20: 4n-6	1.64±0.09^b^	1.24±0.19^bcd^	0.91±0.11^cd^	0.83±0.15^b^	2.97±0.15^a^	1.54±0.11^bc^	0.000
∑n-6^4^	12.85±1.04^bc^	13.67±0.64^bc^	18.20±1.05^a^	15.09±0.20^ab^	14.06±0.48^bc^	11.30±0.22^c^	0.001
C 18: 3n-3	1.34±0.41^b^	2.79±0.24^b^	3.39±0.29^b^	21.11±0.92^a^	1.68±0.16^b^	1.81±0.09^b^	0.000
C 18: 4n-3	0.36±0.02^d^	0.66±0.09^c^	0.50±0.05^cd^	1.62±0.06^a^	0.57±0.01^cd^	0.94±0.04^b^	0.000
C 20: 5n-3	3.04±0.06^c^	2.87±0.40^cd^	1.82±0.15^d^	1.75±0.17^d^	8.96±0.32^a^	5.99±0.15^b^	0.000
C 22: 5n-3	1.49±0.07^b^	1.35±0.18^b^	0.77±0.11^c^	0.71±0.08^c^	2.90±0.10^a^	1.79±0.03^b^	0.000
C 22: 6n-3	11.59±0.27^bc^	9.28±1.57^c^	7.37±1.35^c^	6.49±1.28^c^	26.32±0.79^a^	14.60±0.79^b^	0.000
∑n-3^5^	17.81±0.77^d^	16.95±1.95^d^	13.84±1.35^d^	31.69±0.73^b^	40.42±0.59^a^	25.13±0.92^c^	0.000
∑PUFA^6^	30.66±1.73^c^	30.63±2.59^c^	32.04±0.84^c^	46.78±0.86^b^	54.49±1.07^a^	36.43±0.74^c^	0.000
∑n-3 LC-PUFA	16.12±0.40^bc^	13.51±2.14^cd^	9.96±1.60^cd^	8.96±1.52^d^	38.18±0.45^a^	22.38±0.85^b^	0.000
∑n-3/∑n-6	1.39±0.08^c^	1.23±0.08^c^	0.77±0.12^d^	2.10±0.04^b^	2.87±0.06^a^	2.23±0.12^b^	0.000
C18:1n-9/∑n-3	1.58±0.12^bc^	1.71±0.25^b^	2.66±0.34^a^	0.86±0.05b^cd^	0.39±0.02^d^	0.85±0.05^cd^	0.000

1 Some fatty acids, of which the contents are minor, trace amount or not detected, such as C22: 0, C24: 0, C14: 1, C20: 2n-6, C20:3n-6, were not listed in the table. *Values in the same row with no common superscripts letters are significantly different (*P*<0.05). nd: non-detected.

2 SFA: saturated fatty acid.

3 MUFA: monounsaturated fatty acid.

4 n-6: n-6 unsaturated fatty acid.

5 n-3: n-3 unsaturated fatty acid.

6 PUFA: polyunsaturated fatty acid.

**Table 6:** Serum fatty acid profiles of juvenile Japanese seabass fed diets with different fatty acid profiles (of total fatty acid)^1^ (means ± S.E.M., n = 3)*.

Fatty acid	PA	SA	OA	LNA	N-3 LC-PUFA	FO	*P* value
C 14: 0	0.93±0.03^c^	1.18±0.06^b^	0.56±0.04^d^	0.56±0.01^d^	0.62±0.01^d^	1.75±0.06^a^	0.000
C 16: 0	22.64±0.40^a^	19.71±0.98^b^	12.27±0.48^c^	14.99±0.42^c^	14.40±0.36^c^	18.57±0.33^b^	0.000
C 18: 0	3.89±0.05^c^	4.71±0.25^b^	2.55±0.15^d^	5.68±0.05^a^	4.18±0.20^bc^	3.47±0.16^c^	0.000
∑SFA^2^	27.46±0.41^a^	25.60±1.21^ab^	15.39±0.67^e^	21.23±0.48^cd^	19.20±0.50^d^	23.80±0.31^bc^	0.000
C 16: 1n-7	7.80±0.38^a^	7.32±0.50^a^	2.35±0.38^c^	2.73±0.08^c^	2.06±0.12^c^	5.11±0.18^b^	0.000
C 18: 1n-9	31.72±0.78^b^	28.02±1.54^b^	39.07±1.22^a^	28.09±0.61^b^	12.37±0.33^d^	18.26±0.46^c^	0.000
C 20: 1n-9	1.17±0.05^cd^	1.10±0.04^d^	4.90±0.06^a^	1.19±0.06^cd^	1.38±0.07^c^	2.04±0.04^b^	0.000
C 22: 1n-11	tr	tr	tr	tr	0.31±0.04^b^	0.83±0.07^a^	0.000
C 22: 1n-9	nd	nd	4.10±0.03	nd	nd	nd	
∑MUFA^3^	40.69±0.86^b^	36.44±1.98^bc^	50.41±1.68^a^	32.01±0.58^c^	16.02±0.15^e^	26.24±0.55^d^	0.000
C 18: 2n-6	8.37±0.41^bc^	7.94±0.23^c^	15.63±0.41^a^	9.63±0.36^b^	5.94±0.07^d^	6.37±0.11^d^	0.000
C 20: 4n-6	0.80±0.07^cd^	0.99±0.22^c^	0.42±0.02^d^	0.74±0.06^cd^	3.99±0.01^a^	1.96±0.01^b^	0.000
∑n-6^4^	9.17±0.46^bc^	8.93±0.29^bc^	16.05±0.43^a^	10.37±0.30^b^	9.90±0.06^b^	8.33±0.12^c^	0.000
C 18: 3n-3	0.37±0.04^d^	1.51±0.21^bc^	2.26±0.06^b^	11.24±0.32^a^	0.80±0.07^cd^	1.18±0.04^cd^	0.000
C 18: 4n-3	0.24±0.01^c^	0.51±0.05^b^	0.61±0.03^b^	1.99±0.04^a^	0.25±0.03^c^	0.52±0.01^b^	0.000
C 20: 5n-3	2.65±0.17^c^	3.19±0.31^c^	1.71±0.06^d^	2.80±0.12^c^	8.97±0.09^a^	7.20±0.19^b^	0.000
C 22: 5n-3	1.23±0.08^cd^	1.44±0.11^bc^	0.66±0.01^e^	0.99±0.03^de^	2.05±0.09^a^	1.75±0.04^ab^	0.000
C 22: 6n-3	10.80±0.86^cd^	13.73±0.82^c^	7.78±0.82^d^	13.49±0.82^c^	35.88±0.35^a^	25.03±0.13^b^	0.000
∑n-3^5^	15.29±1.12^e^	20.37±1.26^d^	12.99±0.96^e^	30.51±0.66^c^	47.96±0.55^a^	35.68±0.34^b^	0.000
∑PUFA^6^	24.46±1.34^c^	29.31±1.52^c^	29.05±1.39^c^	40.88±0.43^b^	57.88±0.51^a^	44.01±0.76^b^	0.000
∑n-3 LC-PUFA	14.68±1.09^c^	18.36±1.17^c^	10.16±0.90^d^	17.28±0.95^c^	46.90±0.47^a^	33.98±0.32^b^	0.000
∑n-3/∑n-6	1.67±0.13^e^	2.28±0.08^d^	0.81±0.04^f^	2.95±0.14^c^	4.83±0.08^a^	4.28±0.04^b^	0.000
C18:1n-9/∑n-3	2.10±0.19^b^	1.39±0.16^c^	3.03±0.32^a^	0.92±0.04^cd^	0.26±0.01^d^	0.51±0.01^d^	0.000

1 Some fatty acids, of which the contents are minor, trace amount or not detected, such as C22: 0, C24: 0, C14: 1, C20: 2n-6, C20:3n-6, were not listed in the table. *Values in the same row with no common superscripts letters are significantly different (*P*<0.05). tr: trace; nd: non-detected.

2 SFA: saturated fatty acid.

3 MUFA: monounsaturated fatty acid.

4 n-6: n-6 unsaturated fatty acid.

5 n-3: n-3 unsaturated fatty acid.

6 PUFA: polyunsaturated fatty acid.

**Table 7:** Gut acid profiles of juvenile Japanese seabass fed diets with different fatty acid profiles (of total fatty acid)^1^ (means ± S.E.M., n = 3)*.

Fatty acid	PA	SA	OA	LNA	N-3 LC-PUFA	FO	*P* value
C 14: 0	3.30±0.20^b^	3.97±0.20^ab^	1.19±0.14^c^	1.19±0.14^c^	1.48±0.17^c^	4.55±0.20^a^	0.000
C 16: 0	24.49±1.44^a^	18.46±0.60^b^	9.65±0.45^c^	10.35±0.30^c^	9.41±0.68^c^	16.75±0.41^b^	0.000
C 18: 0	20.82±1.01^a^	16.98±0.60^b^	tr	16.87±0.80^b^	4.10±0.56^c^	2.96±0.16^c^	0.000
∑SFA^2^	50.27±2.21^a^	39.41±1.34^b^	10.84±0.58^d^	28.41±0.59^c^	14.99±0.66^d^	24.27±0.37^c^	0.000
C 16: 1n-7	6.28±0.52^a^	7.07±0.36^a^	2.44±0.16^b^	2.74±0.28^b^	3.41±0.28^b^	7.72±0.08^a^	0.000
C 18: 1n-9	11.95±0.94^c^	11.98±0.94^c^	41.12±0.58^a^	13.66±0.98^c^	12.58±1.18^c^	20.72±0.41^b^	0.000
C 20: 1n-9	3.22±0.30^b^	2.85±0.37^bc^	5.23±0.23^a^	1.25±0.10^d^	2.00±0.13^cd^	3.19±0.12^b^	0.000
C 22: 1n-11	1.83±0.41^ab^	1.63±0.25^ab^	tr	0.64±0.12^b^	0.92±0.26^b^	2.44±0.12^a^	0.006
C 22: 1n-9	nd	nd	7.84±0.40	nd	nd	nd	
∑MUFA^3^	23.27±0.72^c^	23.53±1.01^c^	56.62±0.87^a^	18.30±1.09^d^	18.90±1.57^cd^	34.07±0.51^b^	0.000
C 18: 2n-6	10.00±0.69^c^	13.30±1.36^bc^	18.94±0.18^a^	14.78±0.25^b^	12.94±0.38^bc^	12.46±1.12^bc^	0.000
C 20: 4n-6	0.97±0.06^b^	0.83±0.08b^c^	0.23±0.03^d^	0.23±0.04^d^	2.17±0.05^a^	0.71±0.03^c^	0.000
∑n-6^4^	10.97±0.69^c^	14.13±1.30^bc^	19.18±0.20^a^	15.01±0.22^b^	15.11±0.43^b^	13.18±1.09^bc^	0.001
C 18: 3n-3	1.18±0.11^d^	2.82±0.25^c^	4.92±0.11^b^	29.09±0.55^a^	1.98±0.08^cd^	2.39±0.23^cd^	0.000
C 18: 4n-3	0.52±0.07^d^	0.99±0.08^c^	0.96±0.04^c^	2.10±0.13^a^	0.99±0.02^c^	1.73±0.08^b^	0.000
C 20: 5n-3	1.18±0.18^cd^	2.06±0.36^c^	0.74±0.07^d^	0.89±0.05^cd^	11.72±0.46^a^	5.80±0.10^b^	0.000
C 22: 5n-3	0.80±0.16d^cd^	1.38±0.08^bc^	0.31±0.05^d^	0.40±0.03^d^	3.37±0.22^a^	1.76±0.11^b^	0.000
C 22: 6n-3	4.47±1.08^c^	5.59±0.96^bc^	1.79±0.15^c^	1.84±0.18^c^	25.06±1.08^a^	8.44±0.74^b^	0.000
∑n-3^5^	8.14±1.18^d^	12.84±1.68^d^	8.72±0.18^d^	34.32±0.40^b^	43.12±1.37^a^	20.12±0.68^c^	0.000
∑PUFA^6^	19.11±0.71^e^	26.97±0.78^d^	27.90±0.06^d^	49.33±0.58^b^	58.23±1.80^a^	33.30±0.82^c^	0.000
∑n-3 LC-PUFA	6.44±1.21^cd^	9.03±1.39^c^	2.84±0.27^d^	3.13±0.25^d^	40.15±1.31^a^	16.00±0.85^b^	0.000
∑n-3/∑n-6	0.76±0.15^d^	0.95±0.21^d^	0.45±0.01^d^	2.29±0.02^a^	2.85±0.01^a^	1.55±0.16^b^	0.000
C18:1n-9/∑n-3	1.79±0.21^b^	0.94±0.04^c^	4.72±0.15^a^	0.40±0.03^d^	0.29±0.03^d^	1.03±0.05^c^	0.000

1 Some fatty acids, of which the contents are minor, trace amount or not detected, such as C22: 0, C24: 0, C14: 1, C20: 2n-6, C20:3n-6, were not listed in the table *Values in the same row with no common superscripts letters are significantly different (*P*<0.05). tr: trace; nd: non-detected.

2 SFA: saturated fatty acid.

3 MUFA: monounsaturated fatty acid.

4 n-6: n-6 unsaturated fatty acid.

5 n-3: n-3 unsaturated fatty acid.

6 PUFA: polyunsaturated fatty acid.

The contents of saturated fatty acid (SFA), monounsaturated fatty acid (MUFA) and 18C polyunsaturated fatty acids generally reflected those of the diets. The content of the characteristic fatty acid of each group was higher than that in other groups respectively. Especially, the contents of C18:3n-3 in tissues of fish fed LNA was much higher than those in other groups. Meanwhile, significant positive correlations were observed either between tissue C18:4n-3 contents and tissue C18:3n-3 contents (the correlation coefficient (r) = 0.982, 0.894, 0.990, and 0.741, for liver, muscle, serum, and gut, respectively; *P* = 0.000) or between tissue C18:4n-3 contents and the dietary C18:3n-3 content (r = 0.996, 0.889, 0.982, and 0.745, for liver, muscle, serum, and gut, respectively; *P* = 0.000). However, no significant correlation was observed between tissue C18:4n-3 contents and the dietary C18:4n-3 content (r and *P* was −0.307 and 0.215, −0.026 and 0.918, 0.336 and 0.187, and 0.170 and 0.500, for liver, muscle, serum, and gut, respectively).

### Cloning and characterization of FADS2

**Figure 2:**
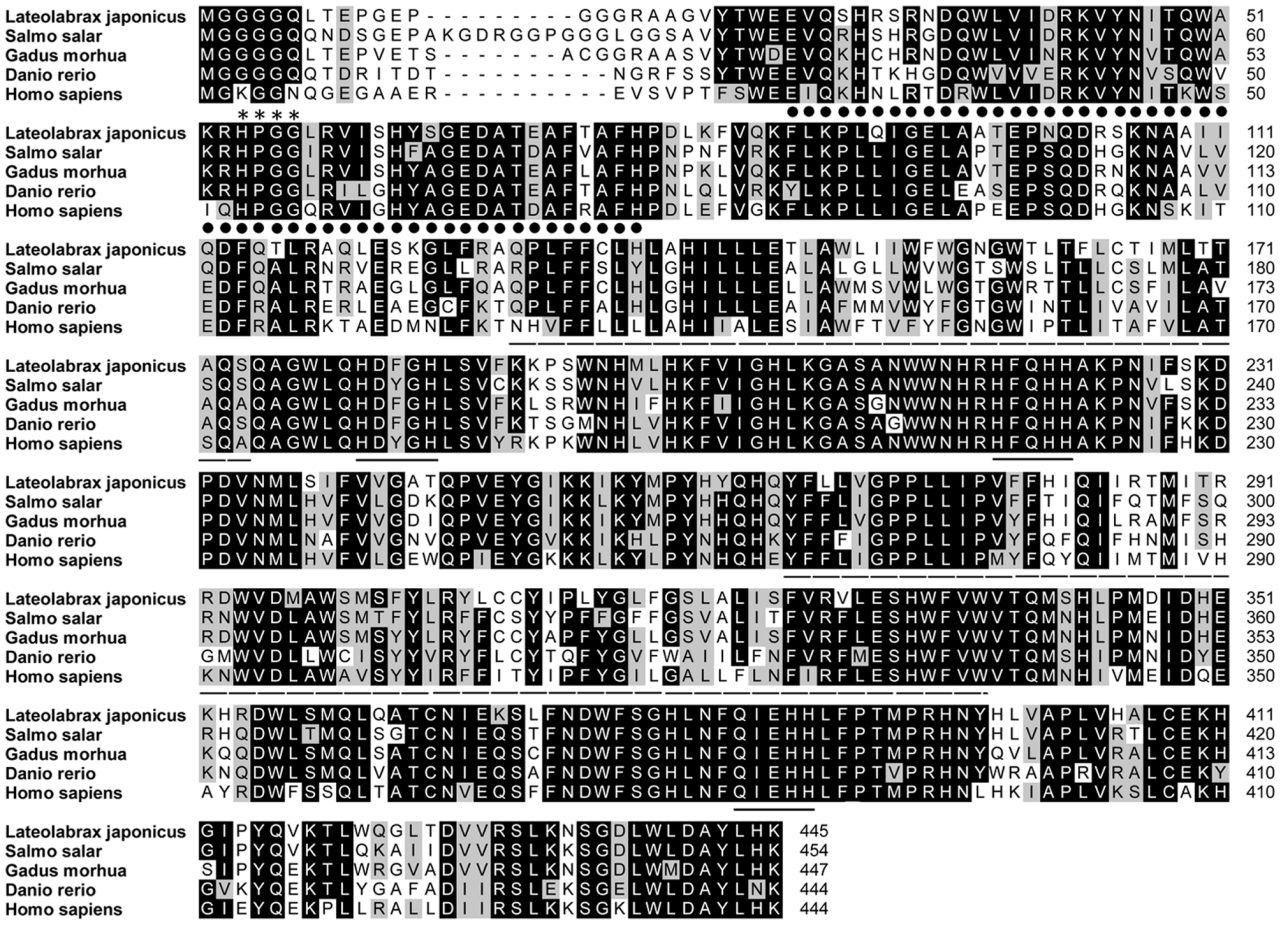
Comparison of the deduced amino acid sequences of FADS2 from Japanese seabass and other fish. The amino acid sequences were aligned using ClustalX, and identity/similarity shading was based on the BLOSUM62 matrix and the cut off was 75%. Identical residues are shaded black and similar residues are shaded grey. The cytochrome *b*5-like domain is dot-underlined, the two transmembrane regions are dash underlined, the three histidine-rich domains are solid underlined and asterisks on the top mark the haem-binding motif, H–P–G–G.

**Figure 3:**
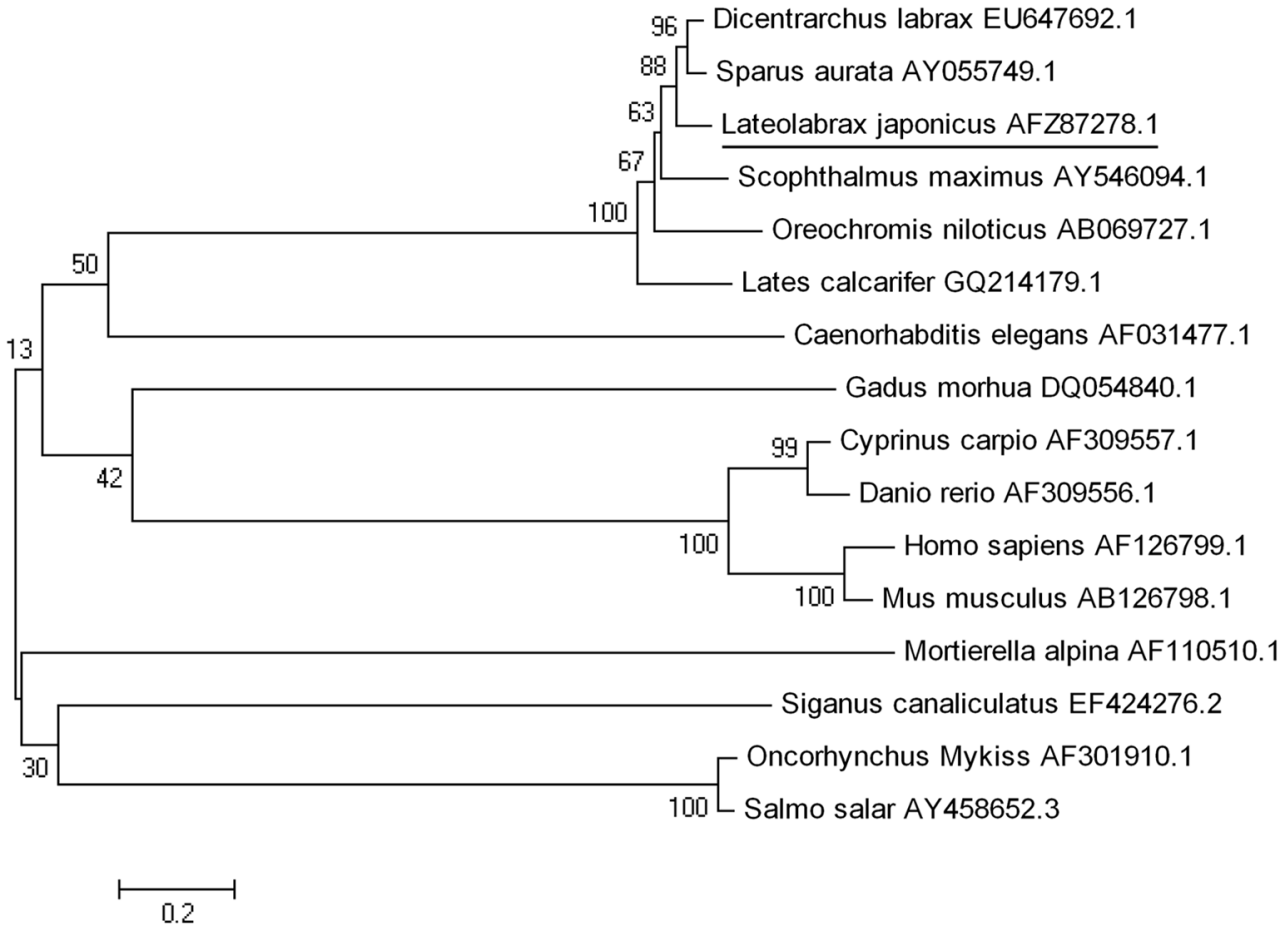
Phylogenetic tree of FADS2. The amino acid sequences of FADS2 used in the phylogenetic tree included those from European sea bass, gilthead sea bream, salmon, zebrafish, other fish species (Atlantic cod, turbot, rabbitfish, barramundi, carp, rainbow trout, and tilapia), mammals (mouse and human), fungus (*M. alpina*) and nematode (*C. elegans*). The horizontal branch length is proportional amino acid substitution rate per site. The numbers represent the frequencies with which the tree topology presented here was replicated after 1000 bootstrap iterations.

**Figure 4:**
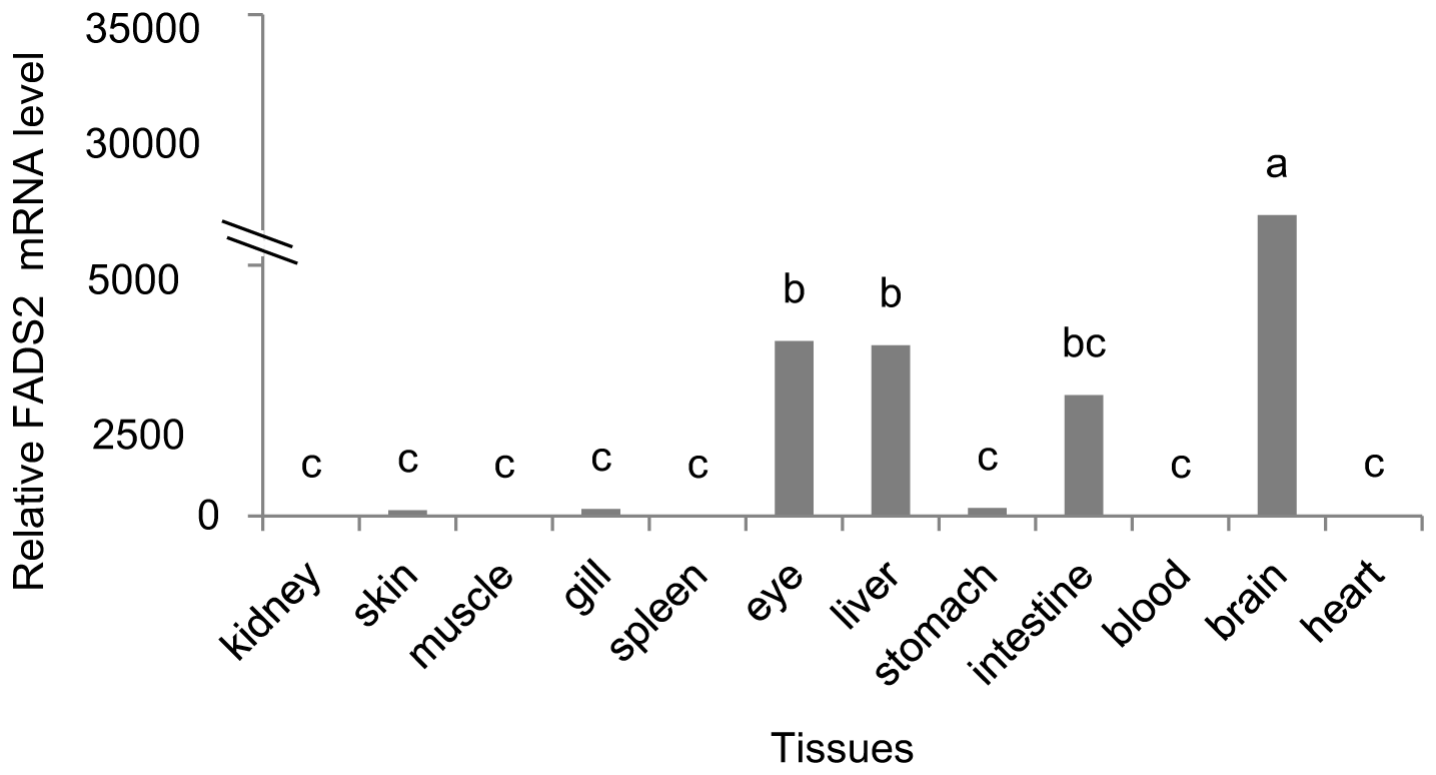
Tissue distribution of FADS2 in Japanese seabass. Relative FADS2 mRNA expression was determined by quantitative real-time PCR (qRT-PCR) as described in the Materials and Methods section. Results are expressed as means ± S.E.M. (n = 3). Different letters above the bars denote significant differences among tissues at the *P*<0.05 level (*P* = 0.000) as determined by one-way ANOVA followed by Tukey's test (SPSS).

The full length of the FADS2 cDNA was 1920 bp (GeneBank accession number: JX678842.1), which included a 5′-UTR (untranslated region) of 143 bp, a 3′-UTR of 439 bp and an open reading frame of 1338 bp encoding a polypeptide of 445 amino acids (GeneBank accession number: AFZ87278.1) with predicted molecular weight of 51.8 kDa and theoretical isoelectric point of 8.86 (Figure 2). The protein sequence possessed all the characteristic features of microsomal fatty acid desaturases, including two transmembrane regions, three histidine boxes and an N-terminal cytochrome *b_5_* domain containing the haem-binding motif, H–P–G–G (Figure 2). The BLAST analysis revealed that the FADS2 of Japanses seabass shared high identity to known FADS2s in marine teleosts, European sea bass (91%), gilthead sea bream (89%), orange-spotted grouper (88%), and cobia (86%). Consistent with this, the phylogenetic analysis showed that Japanese seabass FADS2 was clustered closest to FADS2s of marine fish such as European sea bass and gilthead sea bream, and distant from FADS2s from freshwater fish, and further still from FADS2s from mammals (Figure 3). The real-time PCR assay showed that the gene expression of FADS2 in brain, eye, liver and intestine was significantly higher compared to other tissues such as kidney, skin, muscle, gill, spleen, stomach, blood, and heart (Figure 4).

### FADS2 mRNA expression in response to dietary fatty acids

**Figure 5:**
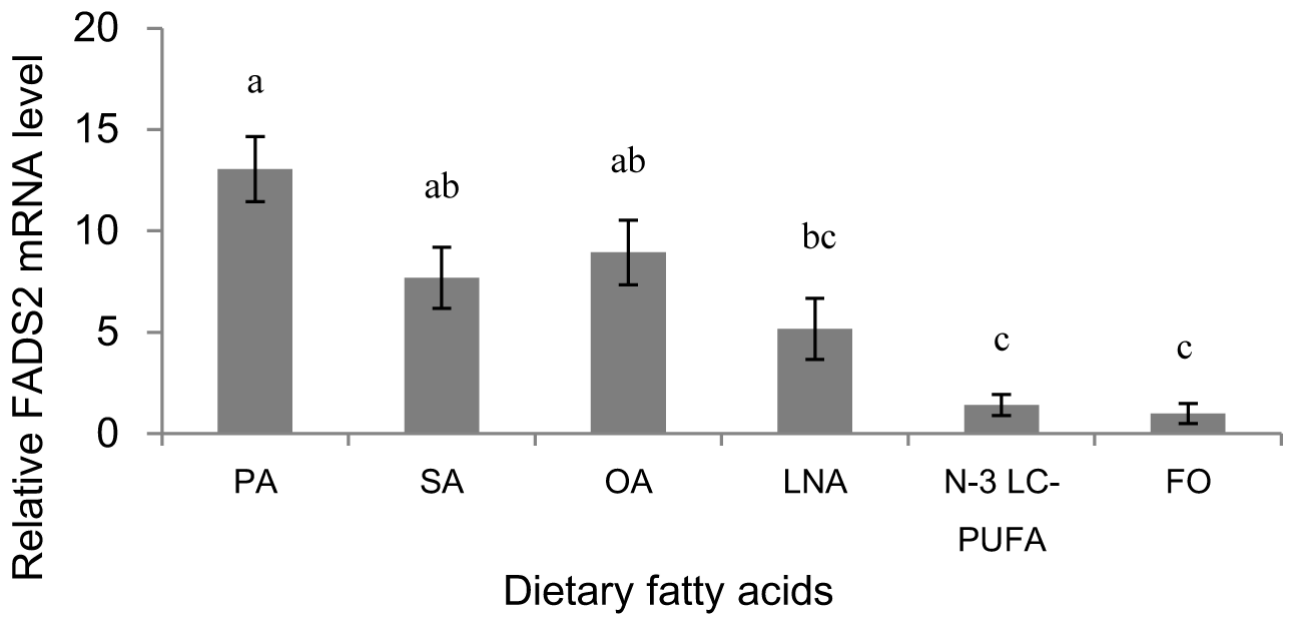
Relative FADS2 mRNA levels in liver of experimental fish. Relative FADS2 mRNA levels were evaluated by quantitative real-time PCR (qRT-PCR) and expressed relative to β-actin levels. Results are expressed as means ± S.E.M. (n = 3). Different letters above the bars denote significant differences among dietary groups at the *P*<0.05 level (*P* = 0.000) as determined by one-way ANOVA followed by Tukey's test (SPSS).

The expression levels of FADS2 transcript were significantly (*P*<0.01) up-regulated in liver of Japanese seabass fed PA compared with fish fed LNA, N-3 LC-PUFA and FO (Figure 5). Fish fed Diet N-3 LC-PUFA and FO showed significantly (*P*<0.01) lower mRNA expression of FADS2 compared to other groups except group LNA.

### Cloning and sequence analysis of FADS2 promoter

**Figure 6:**
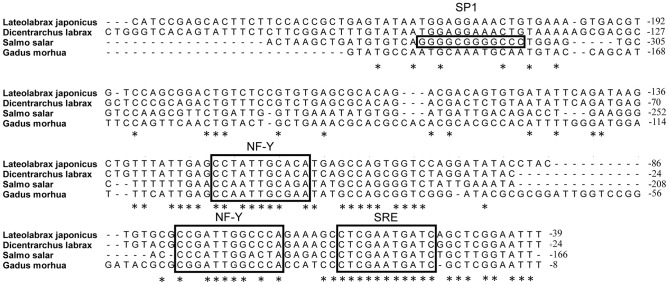
Alignment of FADS2 promoter fragments among Japanese seabass and other fish. The numbers indicate the sequence position relative to the possible transcriptional start site. Binding sites of SP1 and NF-Y, and sterol regulatory element (SRE) were indicated based on previous work from Zheng et al. [44] and Geay et al. [36].

A sequence of 2064 bp upstream from the translation start codon of FADS2 was cloned. This sequence had a homology of more than 73% with the reported counterpart sequence in European sea bass *Dicentrarchus labrax*. Alignments of FADS2 promoter fragments of Japanese seabass, European sea bass, Atlantic salmon, and Atlantic cod, including potential elements of transcription factors (specificity protein 1 (Sp1), nuclear factor Y (NF-Y), and SREBPs), showed that the FADS2 promoter of Japanese seabass possesses elements of NF-Y and SREBPs as European sea bass, Atlantic salmon, and Atlantic cod but lacks Sp1 element which Atlantic salmon possesses (Figure 6).

### The CpG methylation in response to dietary fatty acids

**Figure 7:**
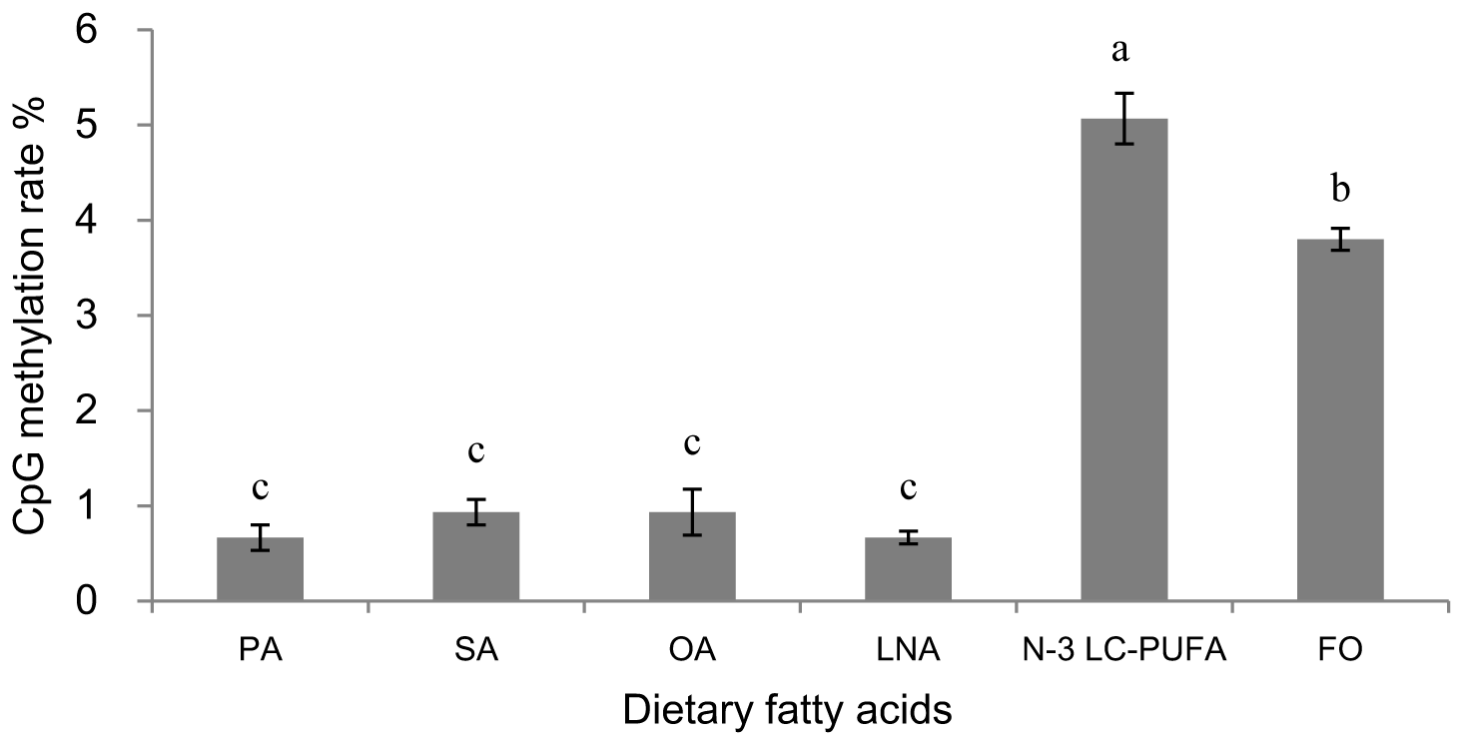
Methylation rate of 55 CpG in FADS2 gene promoter of Japanese seabass. The methylation rate was determined using BSP cloning-based sequencing analysis. Results are expressed as means ± S.E.M. (n = 3). Different letters above the bars denote significant differences among dietary groups at the *P*<0.05 level (*P* = 0.000) as determined by one-way ANOVA followed by Tukey's test (SPSS).

Two CpG islands (CpG island 1: CpG site 1–13; CpG island 2: CpG site 14–55) were found around the transcriptional start site between the upstream −345 bp and downstream +478 bp. The methylated CpG sites were not in putative binding sites of transcription factors such as Sp1, NF-Y, and SREBPs. The determination of the methylation status of 55 CpG sites showed that fish fed N-3 LC-PUFA and FO showed significantly (*P*<0.01) higher methylation rates in FADS2 gene promoter compared to other groups (Figure 7). The methylation rate of FADS2 gene promoter in group N-3 LC-PUFA was significantly higher than that in group FO while no significant difference was observed among group PA, SA, OA and LNA. Significant negative correlations were observed between the CpG methylation rate in FADS2 gene promoter and the liver mRNA expression level of FADS2 (r was −0.814 and *P* was 0.049).

## Discussion

In the present study, treatments with diets enriched with C16:0, C18:0, C18:1n-9, and C18:3n-3 significantly reduced the n-3 LC-PUFA contents in tissues of Japanese seabass, indicating that the euryhaline species Japanese seabass reared in seawater has insufficient capacity to biosynthesize LC-PUFAs and the alternative LC-PUFA-free dietary lipids could cause serious LC-PUFA reduction in fish tissues. This was similar with strict marine species of which the limited capacity of LC-PUFA biosynthesis has been well demonstrated [20], [23], [25], [39]. Being reared in seawater could partially contribute to the low capacity of LC-PUFA biosynthesis in euryhaline Japanese seabass. When reared in seawater, other euryhaline species such as anadromous salmonids, rabbitfish and barramundi have also been reported to display a similar pattern to strict marine fish in capacity to synthesize LC-PUFA [12], [19], [40], [41], [42]. It has been well demonstrated that the desaturation metabolism for LC-PUFA synthesis was regulated by the ambient salinity, with up-regulation by lower salinity [10], [11], [13], [43]. The mRNA expression of FADS2 in liver of rabbitfish reared in 10 ppt salinity was much higher than that in fish reared in 32 ppt salinity, irrespective of diet [12].

Beside the significant difference in n-3 LC-PUFA content between n-3 LC-PUFA deficient groups and n-3 LC-PUFA rich groups, significant differences in n-3 LC-PUFA content were also observed either among n-3 LC-PUFA deficient groups or between the two n-3 LC-PUFA rich groups in this study. Among the LC-PUFA deficient groups (group PA, SA, OA and LNA), the n-3 LC-PUFA contents in muscle and gut of fish fed OA and LNA was lower compared to fish fed PA and SA. This indicated that though the capacity to synthesize LC-PUFAs in Japanese seabass was limited, it functionally existed, since the dietary n-3 LC-PUFA inputs of the LC-PUFA deficient groups (group PA, SA, OA and LNA) were the same and thus the differences in n-3 LC-PUFA content in fish tissues among these groups were probably attributed to the different amounts of synthesized n-3 LC-PUFA in fish regulated by different dietary fatty acid profiles. The functional existence of LC-PUFA synthesis in marine and euryhaline species has been widely demonstrated, though this synthesis was commonly limited. In the present study, significant positive correlations were observed either between tissue C18:4n-3 contents and tissue C18:3n-3 contents or between tissue C18:4n-3 contents and the dietary C18:3n-3 content, but not between tissue C18:4n-3 contents and the dietary C18:4n-3 content. To some extent, this at least indicated the existence of the first step of LC-PUFA biosynthetic pathway, i.e., the desaturation from C18:3n-3 to C18:4n-3.

Moreover, for the two n-3 LC-PUFA rich groups (group N-3 LC-PUFA and FO), the n-3 LC-PUFA contents in group N-3 LC-PUFA was significantly higher than those in group FO in muscle, serum and gut whereas it followed an opposite pattern in liver. Since the n-3 LC-PUFA content in Diet N-3 LC-PUFA (45.18% of TFA) was nearly three times that of Diet FO (15.84% of TFA), this result was probably due to that n-3 LC-PUFAs in muscle, gut and serum were dominated by their direct dietary incorporation but the n-3 LC-PUFA in liver, one of the main organs where LC-PUFAs were synthesized, was highly influenced by the in vivo fatty acid synthesis and the much higher n-3 LC-PUFA content in Diet N-3 LC-PUFA compared to Diet FO inhibited the LC-PUFA synthesis in liver of group N-3 LC-PUFA.

Besides the fatty acid composition, the FADS2 gene expression in liver of Japanese seabass in this study was also regulated by different dietary fatty acid profiles. As stated above, FADS2 was a critical enzyme catalyzing the first and rate-limiting step in the biosynthesis of LC-PUFA from 18:3n-3 and 18:2n-6, and thus was commonly used as the indicator of LC-PUFA biosynthesis. Japanses seabass FADS2 shared high identity to known FADS2s in marine teleosts and the phylogenetic analysis showed that the FADS2 in Japanese seabass clustered closest with marine species European sea bass and gilthead sea bream, which probably contribute to the similarity in LC-PUFA synthesis capacity between Japanese seabass and strict marine fish species. In addition, the lack of binding sites for Sp1 transcription factor in FADS2 gene promoter of Japanese seabass could also lead to a less active promoter, since in salmonids the Sp1 binding site has been shown to be essential for the activity of FADS2 promoter and required for full expression of FADS2 gene [44], [45]. The lack of Sp1 binding sites in European sea bass and Atlantic cod in comparison to salmonids have been observed to be correlated with lower FADS2 gene expressions [36], [44]. The tissue profile of gene expression for FADS2 in Japanese seabass showed that the gene expression of FADS2 in brain and eye was significantly higher compared to other tissues, indicating a role for this enzyme in maintaining high DHA levels in neural and visual tissues through conversion of 20:5n-3, which was confirmed in several previous studies [46], [47]. Regarding the regulation of FADS2 gene expression by dietary fatty acids in the present study, diets enriched with C16:0, C18:0 and C18:1n-9 respectively resulted in significantly higher FADS2 gene expression in liver of Japanese seabass compared with diets rich in LC-PUFA. This was in accordance with results of a number of studies on marine fish or euryhaline fish reared in seawater [12], [13], [43], [48], [49], [50], [51], [52], [53], which showed that the alternative LC-PUFA-free lipids significantly increased the FADS2 gene expression compared to fish oil. It has remained unclear whether the nutritional regulation of FADS2 is due more to desaturation product reduction or to increased substrate supply, or if both are involved. In the present study, it seemed that the FADS2 gene expression was more strongly regulated by FADS2 substrate supply, since though the dietary n-3 LC-PUFA content in group N-3 LC-PUFA was 3-fold of that in group FO, there was no significant difference in liver FADS2 gene expression between these two groups. Some authors also demonstrated that the rate of C18 fatty acids desaturation was more strongly regulated by FADS2 substrates than by DHA [54], [55], [56]. However, Tocher et al. [40] speculated that this rate of desaturation is a direct result of product reduction rather than an increased supply of precursors. Other conclusions that both product reduction and increased substrate supply were factors determining hepatocyte fatty acid desaturation activity have also been reported [57], [58], [59], [60]. Further investigation is required to identify the exact mechanisms involved.

The present study also showed that among the LC-PUFA deficient treatments, the levels of liver FADS2 gene expression were also significantly different, with the lowest value in the C18:3n-3-enriched group, significant higher value in the C16:0-enriched group and intermediate values in the C18:0-enriched group and the C18:1n-9-enriched group. This result could be explained by that an excess of 18:3n-3 in the diet could also block FADS2 gene transcription, which was also observed in gilthead seabream larvae [52]. However, Li et al. [12] reported that in rabbitfish dietary perilla oil which has a very high C18:3n-3 content lead to a higher FADS2 mRNA level compared to safflower oil which is rich in 18:2n-6. Several previous studies reported that the degree of nutritional regulation of FADS2 activity by alternative dietary lipids depends on the dietary ratio of two FADS2 substrates 18:3n-3/18:2n-6 [18], [40], [52], [61]. However, in the present study the C18:3n-3-enriched group (group LNA) having a dietary 18:3n-3/18:2n-6 of 2.39, which normally favors the conversion of 18:3n-3 into LC-PUFA, showed a significantly lower FADS2 mRNA level than the C16:0-enriched group (group PA) with an inappropriate 18:3n-3/18:2n-6 ratio of 0.13. Thus, there must be other factors influencing the gene expression of FADS2 and further studies are needed to elucidate them.

Furthermore, studies showing that FADS2 gene expression was unaltered by different dietary fatty acid profiles have also been reported. A study on Atlantic cod observed that the expression levels of Δ6 desaturase gene in liver and intestine were barely altered by a dietary vegetable oil blend, in comparison with fish oil [14]. This could be related to the very low expression of FADS2 gene in hepatocytes or enterocytes of Atlantic cod. The nutritional regulation of FADS2 could also vary with the developmental stage of fish, as well as the structures of analyzed genes. Monroig et al. [62] reported that the expression levels of the FADS2_a gene in liver and the FADS2_b gene in intestine were significantly higher in fish fed diets containing vegetable oil compared to fish fed fish oil while no significant differences were found between transcript levels for FADS2_a in intestine, FADS2_b in liver, or FADS2_c in liver or intestine of fish fed different lipid sources.

Though the regulation of FADS2 gene expression by dietary fatty acids has been widely studied, the mechanism involved was poorly understood. Information about binding sites for transcription factors such as SREBPs, NF-Y, and Sp1 have been reported in FADS2 genes in some fish species [44], but no information was available about the regulation of these transcription factors by dietary fatty acids in fish. DNA methylation, another major epigenetic mechanism implicated in the nutritional regulation of FADS2 gene transcription, was also rarely studied in fish with respect to the regulation by dietary fatty acids. In the present study, the methylation of CpG dinucleotides in FADS2 gene promoter was significantly regulated by dietary fatty acid profiles. Dietary treatments with LC-PUFA deficient fatty acid profiles recorded much lower CpG methylation rate in FADS2 gene promoter compared to diets rich in n-3 LC-PUFA. Since fatty acids were not efficient methyl donors, a possible way by which dietary fatty acids affect DHA methylation was affecting the DNA methyltransferases [63] though no information regarding this has been reported up to date. What was more important in this result, a significant negative correlation was observed between the promoter CpG methylation rate and the mRNA expression level of FADS2, suggesting that the regulation of the methylation status of gene promoter by dietary fatty acids probably contributed to the regulation of FADS2 gene expression by dietary fatty acids. Since in this study the methylated CpG sites were not in the putative binding sites of the main lipid metabolism regulating transcription factors such as Sp1, NF-Y, and SREBPs, the possible inhibition of FADS2 gene expression by DNA methylation might not function through the direct inhibition of the binding of these transcription factors to their binding site in the gene promoter. It might function through indirect ways that methylcytosine-binding proteins bound to methylated CpGs form complexes with histone deacetylases and corepressors, leading to histone deacetylation, chromatin condensation and a transcriptionally inactive chromatin structure [63], or that these methylated CpG sites were included in binding sites of other transcription factors regulating this metabolic process. The possible methylation silencing of liver FADS2 expression was also found in the study of Devlin et al. [64] which revealed that mice with FADS2 promoter hypermethylation caused by hyperhomocysteinemia also showed lower FADS2 mRNA (*P*<0.01) and lower Δ6 desaturase activity (*P*<0.001) in liver. However, Geay et al. [36] observed that though the FADS2 gene expression in European sea bass was stimulated by low dietary LC-PUFA contents compared to high LC-PUFA contents, the methylation of FADS2 gene promoter was not influenced by dietary treatments. This absence of significant influences on promoter methylation of European sea bass FADS2 gene by different dietary LC-PUFA contents might be due to that the grade of dietary LC-PUFA contents in this study was not as large as the present study. It also might be due to species and other dietary nutrients. Up to date, the relationship among FADS2 gene expression, methylation status of gene promoter and dietary fatty acids was poorly understood and further research is needed.

In conclusion, when reared in seawater, euryhaline species Japanese seabass displayed a low capacity to biosynthesize LC-PUFA, similar with strict marine species. The n-3 LC-PUFA contents in fish fed diets enriched with C16:0, C18:0, C18:1n-9, and C18:3n-3 respectively were significantly compromised compared to fish fed diets enriched with n-3 LC-PUFA and fish oil. The mRNA expression of FADS2 in liver was up-regulated by diets enriched with C16:0, C18:0 and C18:1n-9 respectively compared to fish fed diets with high n-3 LC-PUFA contents and among the dietary groups with deficient LC-PUFA, the diet enriched with C16:0 resulted in significantly higher FADS2 gene expression than the diet enriched with C18:3n-3. The FADS2 gene expression regulated by dietary fatty acid profiles was significantly negatively correlated with the CpG methylation rate in FADS2 gene promoter of Japanese seabass. These findings provide further insight into the nutritional regulation of LC-PUFA biosynthesis in euryhaline fish.
